# Lgr5+ telocytes are a signaling source at the intestinal villus tip

**DOI:** 10.1038/s41467-020-15714-x

**Published:** 2020-04-22

**Authors:** Keren Bahar Halpern, Hassan Massalha, Rachel K. Zwick, Andreas E. Moor, David Castillo-Azofeifa, Milena Rozenberg, Lydia Farack, Adi Egozi, Dan R. Miller, Inna Averbukh, Yotam Harnik, Noa Weinberg-Corem, Frederic J. de Sauvage, Ido Amit, Ophir D. Klein, Michal Shoshkes-Carmel, Shalev Itzkovitz

**Affiliations:** 1 Department of Molecular Cell Biology, Weizmann Institute of Science, 7610001 Rehovot, Israel; 2 Program in Craniofacial Biology and Department of Orofacial Sciences, University of California, San Francisco, CA 94110 USA; 3 Institute of Molecular Cancer Research, University of Zurich, 8002 Zurich, Switzerland; 4 Department of Developmental Biology and Cancer Research, The Institute for Medical Research Israel-Canada, The Hebrew University Hadassah Medical School, 9112001 Jerusalem, Israel; 5 Department of Molecular Oncology, Genentech, San Francisco, CA 94080 USA; 6 Department of Immunology, Weizmann Institute of Science, 7610001 Rehovot, Israel; 7 Department of Pediatrics and Institute for Human Genetics, University of California, San Francisco, CA 94110 USA

**Keywords:** Extracellular signalling molecules, Differentiation, Intestinal stem cells, Single-cell imaging

## Abstract

The intestinal epithelium is a structured organ composed of crypts harboring Lgr5+ stem cells, and villi harboring differentiated cells. Spatial transcriptomics have demonstrated profound zonation of epithelial gene expression along the villus axis, but the mechanisms shaping this spatial variability are unknown. Here, we combine laser capture micro-dissection and single cell RNA sequencing to uncover spatially zonated populations of mesenchymal cells along the crypt-villus axis. These include villus tip telocytes (VTTs) that express *Lgr5*, a gene previously considered a specific crypt epithelial stem cell marker. VTTs are elongated cells that line the villus tip epithelium and signal through Bmp morphogens and the non-canonical *Wnt5a* ligand. Their ablation is associated with perturbed zonation of enterocyte genes induced at the villus tip. Our study provides a spatially-resolved cell atlas of the small intestinal stroma and exposes *Lgr5*+ villus tip telocytes as regulators of the epithelial spatial expression programs along the villus axis.

## Abstract (web summary)

Epithelial gene expression has been shown to be zonated along the crypt-villus axis, but mechanisms shaping this spatial variability were unknown. Here, Bahar Halpern et al. uncover zonation of mesenchymal cells, including Lgr5+ telocytes, which regulate epithelial gene expression at the villus tip.

## Introduction

The small intestine is a structured organ composed of repeating crypt-villus units. *Lgr5*+ epithelial stem cells at the base of the crypts continuously divide to give rise to proliferative progenitors that migrate towards the villi^1,2^. As they approach the crypt exits, these progenitors become post-mitotic and differentiate into distinct lineages–absorptive enterocytes, mucus-secreting goblet cells, enteroendocrine cells and tuft cells. The differentiated cells operate for about three days as they continue to migrate along the villus walls until they are shed from the villi tips^3^.

Villi cells have traditionally been considered ‘terminally-differentiated’ in the sense that they have irreversibly committed to a functional cell state, which they carry from birth to death. Recent studies challenged this view and demonstrated that villi epithelial cells constantly change their functional state, such that around 85% of enterocyte genes are expressed in a non-uniform manner along the villus axis^4^. Enterocytes first implement anti-microbial programs at the base of the villi, then shift to sequential absorption of amino acids, carbohydrates and lipids in distinct villi zones, finally upregulating genes associated with cell adhesion and immune modulation at the villi tips. Similarly, enteroendocrine cells change the types of hormones they produce as a function of their position along the villus axis^5,6^. The epithelial lineages along the intestinal villus therefore exhibit profound spatial heterogeneity.

What are the mechanisms that facilitate the spatial heterogeneity of the villus epithelium? One mechanism could be an internal ‘clock’, whereby epithelial cells are pre-programmed to turn distinct functions on and off at different times. A second mechanism could be a zonated response to spatial gradients of nutrients and bacteria at the luminal sides of the tissue. Yet a third mechanism could entail zonated molecular cues that originate in the lamina propria, the underlying stroma at the basal sides of the epithelial layer^7–11^. Here, we use spatially-resolved transcriptomics to characterize the zonated stromal gene expression signatures along the crypt-villus axis. We identify four mesenchymal cell populations residing at distinct crypt-villus zones. These include a sub-population of telocytes localized at the villus tip that is marked by *Lgr5*, a gene previously considere a specific marker of epithelial crypt stem cells. We suggest that *Lgr5*+ villus tip telocytes may regulate the epithelial gene expression programs at the villus tip.

## Results

### A spatial expression atlas of the small intestinal stroma

**Fig. 1:**
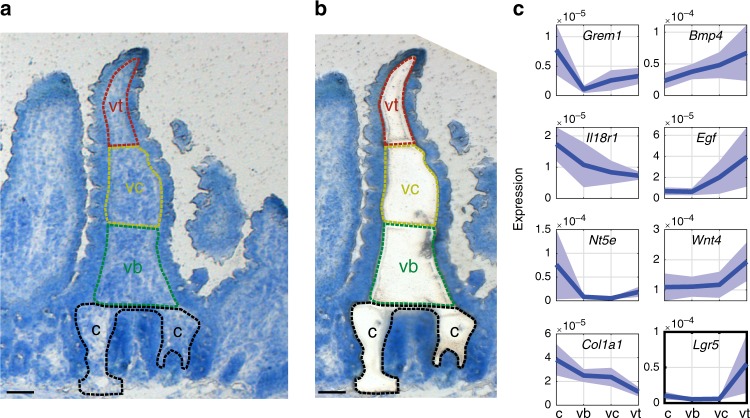
Spatial transcriptomics of the intestinal stroma. **a**–**b** LCM of four zones along the crypt-villus axis before (**a**) and after (**b**) laser dissection. c–crypt, vb–villus bottom, vc–villus center, vt–villus tip. Scale bar–50 µm. **c** Representative spatial LCMseq expression profiles of stromal ligands and receptors zonated towards the crypt (left) or villus tip (right). Units are fraction of sample mRNA, patches are SEM.

To obtain a global view of spatial heterogeneity of stromal gene expression we used Laser Capture Microdissection (LCM) to isolate four stromal zones along the jejunum crypt-villus axis from five mice (Fig. 1a, b). We performed RNA sequencing of these segments, yielding a coarse zonation map (Supplementary data 1). We focused on the zonation patterns of ligands and receptors^12,13^, since these are likely to implement zonated cross-talk with the epithelium (Fig. 1c, Supplementary Fig. 1a, b, Supplementary data 2). Examples of ligand/receptor genes that exhibited higher expression in the crypt stroma included *Grem1*, encoding the BMP pathway inhibitor Gremlin1^14^ (Fig. 1c) and *Il18r1*, the receptor for *Il18*. The cytokine *Il18* has been shown to be expressed in enterocytes at the lower villi zones, as part of an anti-microbial gene module^4^. Using single molecule Fluorescence in-situ Hybridizatoin (smFISH^15^), we identified type 3 innate lymphoid cells (ILC3) expressing *Il18r1* at the stromal side of these zones (Supplementary Fig. 1c–g), suggesting spatial recruitment of these immune cell components by zonated enterocyte *Il18* signal.

### *Lgr5* is abundantly expressed in villus tip stromal cells

We next focused on stromal ligands and receptors that were zonated towards the villi tips in our stromal LCM-RNAseq data. These included *Bmp4*, a morphogen that was shown to inhibit epithelial proliferation^16^ and to control enteroendocrine cell differentiation pathways at the tip^5^ (Fig. 1c). Other villus tip molecules included the non-canonical Wnt ligand *Wnt4* and the epidermal growth factor *Egf. Egfr*, the receptor for *Egf*, has elevated expression levels in villus tip enterocytes^4^, constituting another example of correlated spatial expression of pairs of epithelial and stromal ligands and their matching receptors (Supplementary data 2). We further analyzed zonated proteins constituting the extracellular matrix (ECM, also termed the ‘matrisome’^17^), identifying distinct collagens and matrix metalloproteinases that are differentially zonated between the crypt and villus stroma (Supplementary Fig. 1 h). These included the crypt enriched *Lama2*, encoding the laminin subunit alpha-2 and the villus enriched *Lama5*, encoding the laminin subunit alpha-5, previously shown to be differentially zonated at the protein level^18,19^.

**Fig. 2:**
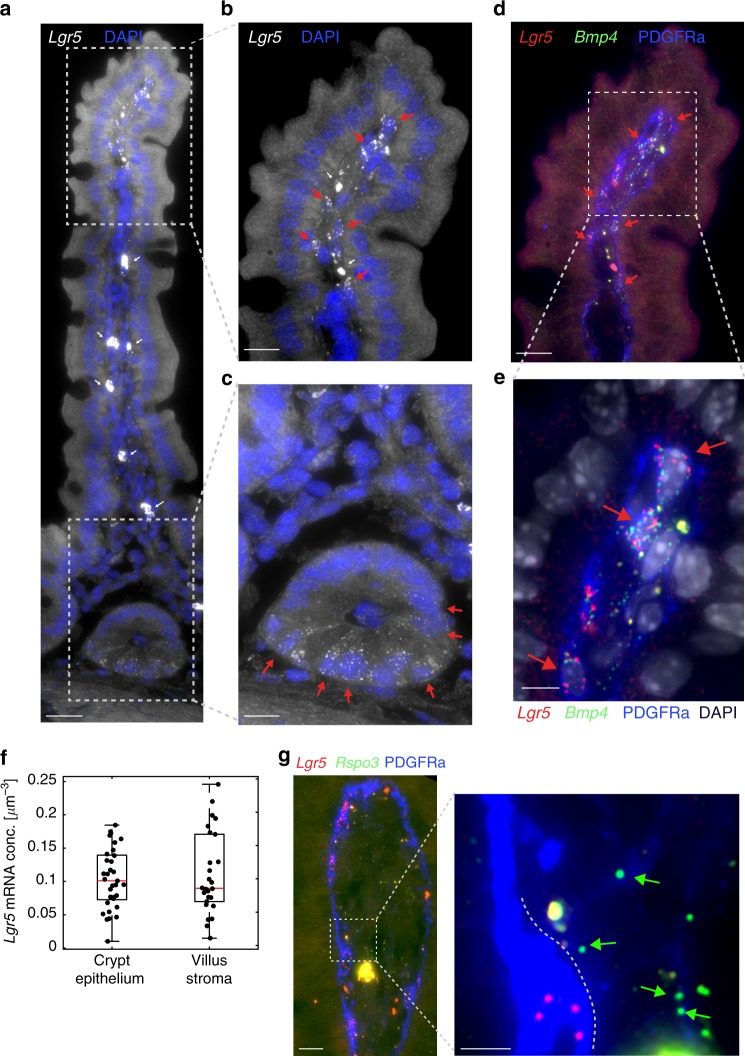
*Lgr5* is expressed abundantly in villus tip telocytes. **a** smFISH of *Lgr5*, DAPI in blue, Scale bar–20 µm. **b** Blow up of villus tip, Scale bar–10 µm. In a, b thin white arrows point at autofluorescent blobs. **c** blow up of crypt, Scale bar–10 µm. Red arrows in **b**–**c** point to *Lgr5* positive cells. **d** *Lgr5* mRNA (red dots) expressed in PDGFRa+ VTTs that co-express *Bmp4* mRNA (green dots). Scale bar–10 µm. Red arrows point to *Lgr5* and *Bmp4* double positive cells. **e** Blow up of the region boxed in **d**. Scale bar–5 µm. **f** *Lgr5* mRNA concentrations in VTTs are comparable to those in *Lgr5*+ crypt base columnar cells (*n* = 25 cells examined over 2 mice for each region). Boxes show 25–75 percentiles of the smFISH expression, horizontal red lines are medians. Whiskers, extend to the most extreme data point within 1.5× the interquartile range (IQR) from the box; **g**) *Rspo3* mRNAs are localized on telopodes that extend away from the cell bodies of the VTTs. VTTs are marked by *Lgr5* mRNA (red dots), *Rspo3* mRNA (green dots) is localized away from the cell body, PDGFRa antibody mark VTTs cell bodies and telopodes. Scale bar–10 µm, in inset, green arrows point to *Rspo3* mRNAs (green dots) localized on PDGFRa telopodes (blue). Telocyte cell body is marked by white dashed line. inset Scale bar–5 µm.

Our zonation analysis revealed *Lgr5* to be one of the most highly expressed receptors in the villus tip stroma (Fig. 1c, Supplementary data 1). *Lgr5* has been shown to be a specific marker of epithelial stem cells at the crypt base^2,15^. It was therefore unexpected to observe elevated expression levels of this gene at the lamina propria of the villus tip. To validate this finding, we performed smFISH and detected abundant localized expression of *Lgr5* transcripts in PDGFRa+ telocytes that co-expressed *Bmp4*, a classic villus tip ligand^20^ (Fig. 2a–e). Telocytes are large mesenchymal cells with elongated extensions that stain positively for the PDGFRa surface marker. They form intricate contacts with both epithelial cells and other stromal cell types^9,21^. Telocytes that surround the crypts constitute important niche cells that secrete canonical Wnt morphogens and Rspo3 to maintain stemness of crypt *Lgr5*+ epithelial stem cells^9,11,22,23^. The molecular identities of telocytes residing in the villi stroma have thus far not been characterized.

The expression levels of *Lgr5* in the villus tip telocytes (VTTs) were comparable to the expression levels in the epithelial crypt base columnar stem cells (mean expression of 0.109 ± 0.012 mRNAs/µm^−3^ in tip telocytes vs. 0.104 ± 0.008 mRNAs/µm^−3^ in crypt stem cells, (Fig. 2f). VTTs expressed both *Lgr5* and its ligand *Rspo3*^24^ (Fig. 2g). Notably, mRNAs of *Rspo3* were localized along telopodes– thin PDGFRa+ extensions of the telocytes that extended towards the lamina propria and away from the cell body, where *Lgr5* mRNAs were localized (Fig. 2g).

**Fig. 3:**
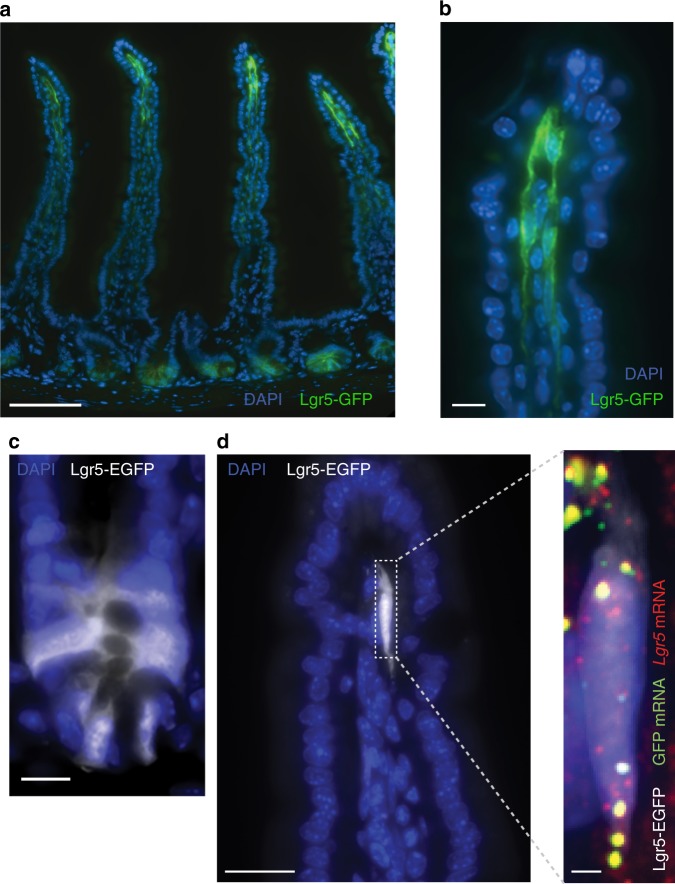
*Lgr5* expression at the villus tip of Lgr5-knock-in mouse models. **a** GFP fluorescence (green) observed in both crypt base columnar cells (CBCs) and VTTs in *Lgr5–GFP-DTR* mice. Scale bar–100 µm. **b** blow up of villus tip with GFP+ VTTs, scale bar–10 µm. **c** EGFP fluorescence of crypt base columnar cells in *Lgr5-EGFP-IRES-creERT2* mice. Scale bar–10 µm. **d** EGFP fluorescence in VTTs. The Lgr5-EGFP knock-in construct is expressed in a patchy manner in the villus tip stroma. Scale bar–20 µm. Blow up shows *Lgr5* mRNAs in red, EGFP mRNA in green and DAPI in blue. Scale bar–2 µm. Yellow blobs are autofluorescent elements.

Several mouse models have been developed to investigate intestinal *Lgr5*+ crypt epithelial stem cells. These include models for ablating *Lgr5* cells^25^ and for tracing their progenies^2^. We asked whether the knock-in constructs in these mice were also expressed in *Lgr5*+ VTTs. Indeed, VTTs were clearly seen in *Lgr5–GFP-DTR* mice^25^ (Fig. 3a, b) and *Lgr5-EGFP-Ires-CreERT2* mice^2^ (Fig. 3c, d). All GFP+ stromal cells had *Lgr5* mRNA in the *Lgr5–GFP-DTR* mice (60 out of 60 cells counted over 10 villi from 2 mice) and no *Lgr5*+ cells at the villus tip stroma lacked GFP. The proportions of *Lgr5*+ VTTs positive for the EGFP knock-in in the *Lgr5-EGFP-Ires-CreERT2* mice were lower than the proportion of EGFP+ crypts (13% ± 3% vs. 35% ± 6%, counted over 22 villi in two mice), indicating a partial silencing of this knock-in reporter in the mesenchymal lineage in this mouse model. In summary, Lgr5 specifically labels telocytes localized at the villus tip, in addition to its documented role as a marker of epithelial crypt stem cells.

### Single cell transcriptomics of villus mesenchymal cells

**Fig. 4:**
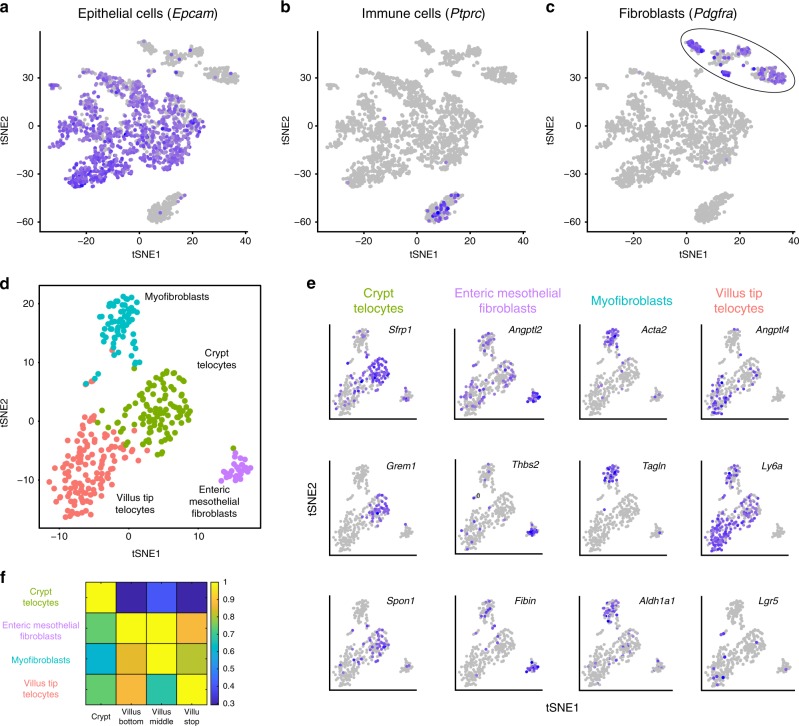
Single cell transcriptomics of the intestinal stroma identifies four spatially-stratified mesenchymal cell populations. **a**–**c** tSNE plots of sequenced single cells highlighting the expression of Epcam, an epithelial marker (**a**), *Ptprc*, encoding CD45, a pan-immune marker (**b**) and *Pdgfra* (**c**) a mesenchymal marker. Purple hue indicates log expression levels. **d** tSNE plot of the Pdgfra+ cells from the cluster circled in **c**. Colors denote the identified four clusters, obtained by re-clustering the Pdgfra+ cluster cells in **c**. **e** tSNE plots colored by selected markers for the four mesenchymal cell clusters (see Supplementary Fig. 3 and Supplementary data 4 for the complete list of markers). *Lgr5* is significantly enriched in the Villus Tip Telocytes (7 out of 10 Lgr5+ cells belong to the VTT cluster, hypergeometric *p* = 0.0121). **f** Summed expression of the top markers for each mesenchymal cell cluster in the LCMseq data, normalized by the maximum across zones (Methods).

We next sought to obtain the global gene expression signatures of Lgr5+ VTTs and of other intestinal mesenchymal cell populations. LCMseq provides a spatial map of gene expression along the crypt villus axis, however each zone represents a mixture of diverse cell types, including mesenchymal cells, endothelial cells, immune cells^26^ and enteric neurons^27^. Unraveling the cellular origins of the zonated transcripts requires single cell transcriptomics. Extracting telocytes is challenging due to their long thin extensions and their entrenchment within the ECM. PDGFRa was previously shown to be expressed in telocytes throughout the crypt-villus axis^9^. We therefore used fluorescence activated cell sorting to enrich for PDGFRa+ cells that included the intestinal telocytes, as well as other mesenchymal cell types (Supplementary Fig. 2). We performed single cell RNA sequencing on these cells using the MARS-seq protocol^28^. Although we enriched for PDGFRa+ cells, our scRNAseq map included predominantly epithelial cells and immune cells, most likely obtained due to the attachment of PDGFRa+ telopode fragments during the dissociation processes. Importantly, our extraction also yielded 329 pure mesenchymal cells (Fig. 4a–c, Methods).

We focused on the mesenchymal cell clusters that were positive for PDGFRa. Reclustering these 329 cells yielded four distinct mesenchymal cell populations (Fig. 4d–e, Supplementary data 3). We used Seurat^29^ to identify distinct gene expression markers for each of the four cell populations (Fig. 4e, Supplementary Fig. 3, Supplementary data 4). To identify potential zonation of these mesenchymal cell types we used our LCMseq map (Supplementary data 1) to examine the expression of the marker genes from each cluster (Fig. 4f, Methods). Using the marker gene identities and their spatial patterns, we annotated the clusters as crypt telocytes, enteric mesothelial fibroblasts, myofibroblasts and villus tip telocytes (VTTs). 10 cells out of the 329 mesenchymal cells were *Lgr5*+, 7 of which were in the VTT cell population (Fig. 4e, hypergeometric *p* = 0.0121). The remaining 3 *Lgr5*+ cells in our scRNAseq data were interspersed with 1 cell in each of the remaining three cell populations.

We used smFISH to validate the spatial patterns of expression of the cell type markers revealed by the scRNAseq measurements. *Grem1* and *Sfrp1* expression was elevated in telocytes at the bottom of the crypts (Supplementary Fig. 4a, c). *Ly6a* and *Angptl4* expression was elevated in VTTs (Supplementary Fig. 4b, c). Notably, although the scRNAseq data suggested higher levels of Bmp2 and Bmp4 ligands in crypt telocytes, our smFISH measurements showed significantly higher levels of *Bmp2* and *Bmp4* in VTTs, in line with the LCM measurements (Fig. 1c, Supplementary Fig. 4c). This inconsistency might stem from the low capture rate of VTT transcripts. Enteric mesothelial fibroblasts^27^ were marked by *Thbs2*, *Fibin* and *Rgs5*, as well as *Angptl2*, a gene previously shown to inhibit Bmp^30^. These were interspersed throughout the crypt-villus axis and were localized at the core of the villus, away from the epithelial layer (Supplementary Fig. 4d), as were myofibroblasts, marked by *Acta2*, *Tagln* and *Aldh1a1* (Fig. 4e).

**Fig. 5:**
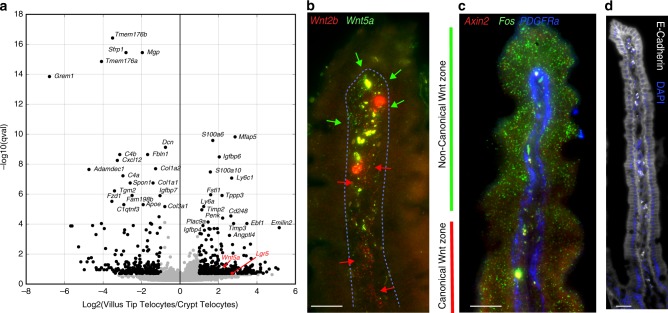
VTTs implement a spatial switch from canonical to non-canonical Wnt signaling. **a** Volcano plot demonstrating differential gene expression between VTTs and crypt telocytes. Black dots have *q*-values < 0.2 and expression fold change larger than 2 or smaller than ½. *Wnt5a* and *Lgr5* are marked in red. **b** Spatial shift in the stroma from the expression of canonical Wnt2b (red dots, marked by red arrows) to non-canonical Wnt5a (green dots, marked by green arrows). Scale bar–20 µm. **c** *Axin2* (red dots) is expressed broadly along the villus axis and repressed at the villus tip. Fos (green dots) is highly expressed in villus tip enterocytes. scale bar–20 µm. **d** E-Cadherin protein (gray), encoded by *Cdh1* gene, is induced in villus tip enterocytes. Large blobs in **b**–**d** are autofluorescent signals originating in immune cells. scale bar–30 µm.

VTTs, as well as crypt telocytes, expressed high levels of ECM components such as *Col3a1*, *Col1a1,* and *Timp2* (Supplementary data 3). VTTs expressed elevated levels of genes encoding microfibrillar proteins such as *Mfap5*, *Emilin2,* and *Fbn1* (Fig. 5a, Supplementary Fig. 3, Supplementary data 3). Differential gene expression between crypt telocytes and VTTs further revealed elevated levels of the non-canonical Wnt ligand *Wnt5a* in VTTs (Fig. 5a,b), suggesting that VTTs implement a switch from canonical to non-canonical Wnt signaling^31,32^. Indeed, we observed broad expression of *Axin2*, a transcriptional target of canonical Wnt signaling, along the villi epithelial cells, with a decrease at the villus tip (Fig. 5c), the zone of stromal *Wnt5a* expression.

### VTT ablation perturbs villus tip epithelial gene expression

**Fig. 6:**
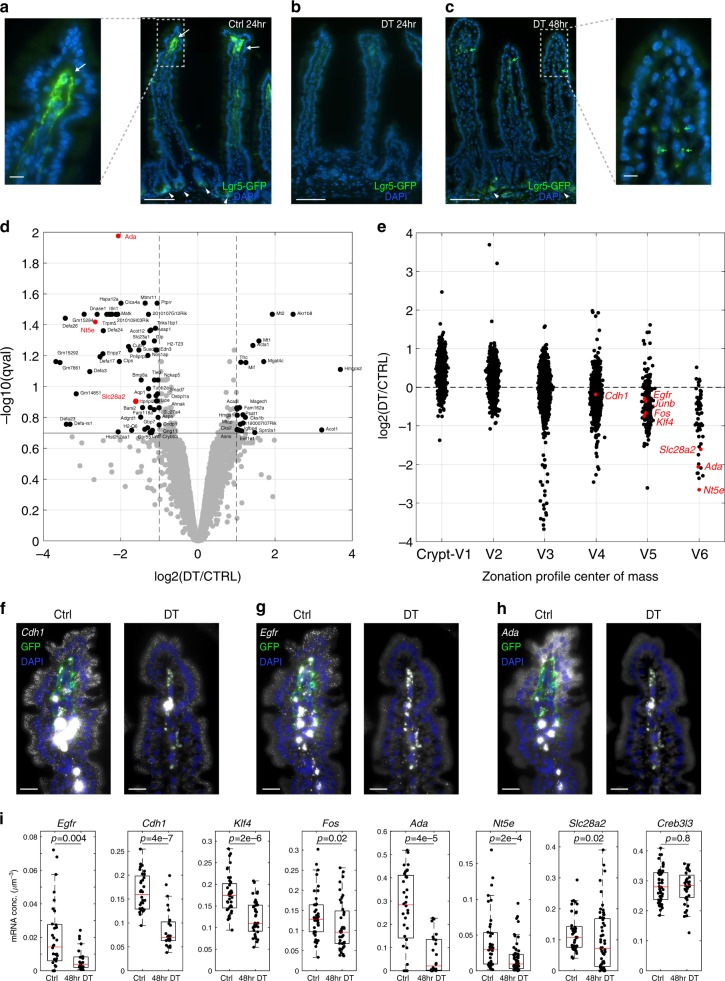
VTT ablation perturbs enterocyte expression at the villus tip. **a** GFP fluorescence (green) observed in both crypt base columnar cells (CBCs, white arrowheads) and VTTs in *Lgr5–GFP-DTR* mice. Inset on left shows a blow up of villus tip with GFP + VTTs (white arrow). **b** Both VTTs and CBCs are ablated 24 h after DT administration, as evident by the lack of GFP fluorescence. **c** GFP fluorescence re-appears in the crypt (white arrowheads) but not at the villus tip stroma 48 h after DT administration, indicating stable loss of VTTs. Inset on right shows a blow up of villus tip with no GFP+ VTTs. Green blobs are autofluorescent elements, also marked by green arrows. Scale bar in **a**–**c** −100 µm, insets scale bar–10 µm. **d** Volcano plot demonstrating the changes in enterocyte gene expression 48 h following VTT ablation. Enterocyte villus tip genes that are reduced include *Ada*, *Nt5e*, and *Slc28a2* (red), composing the purine metabolism immune-modullatory tip module^4^. Black genes have q-values lower than 0.2 and max expression higher than 5 × 10^−6^ (Methods). **e** Enterocyte genes normally induced at the villus tip are reduced in expression 48 h following VTT ablation. Correlation between change in expression and expression zone—*R* = −0.53, *p* < 10^−60^. Enterocytes were classified into six villus zones as in Moor et al.^4^. **f**–**h** smFISH validations of enterocyte villus tip genes that are changed 48 h following VTT ablation—in each panel Ctrl-left, DT-right. **f** *Cdh1*. **g** *Egfr*. **h** *Ada*. Scale bar in **f**–**h** −20 µm. **i** Quantification of smFISH experiments, demonstrating that key epithelial villus tip genes are repressed 48 h after VTT ablation (*Egfr, Cdh1, Klf4, Fos, Ada, Nt5e, Slc28a2 p*-values are indicated), whereas others remain unchanged (Creb3l3), *p*-values were calculated by two-sided Wilcoxon rank sum test. *n* = 30 cells examined over 2 mice. Boxes show 25–75 percentiles of the smFISH expression, horizontal red lines are medians, whiskers extend to the most extreme data point within 1.5× the interquartile range (IQR) from the box.

Enterocytes undergo profound gene expression changes as they approach the villus tip, a few hours before they are shed off into the lumen^4^. The villus tip enterocyte expression program includes induction of the adherens junction protein E-cadherin (Fig. 5d), the AP-1 transcription factor components *Fos* (Fig. 5c) and *Jun*, the transcription factor *Klf4* and the immune-modulatory purine-metabolism program consisting of *Ada*, *Nt5e,* and *Slc28a2*^4^. To assess the role of VTTs in regulating enterocyte zonation at the tip, we ablated *Lgr5* cells using the *Lgr5–GFP-DTR* mouse model^25^, in which *Lgr5*+ cells express diphtheria toxin (DT) receptor and GFP. In this mouse model, 100% of *Lgr5*+ VTTs were positive for GFP (60 out of 60 cells counted over 10 villi from 2 mice, Fig. 3a, b, Fig. 6a, Supplementary Fig. 5a). 24 h after DT administration, *Lgr5*+ VTTs were completely ablated, as were the crypt *Lgr5*+ stem cells (Fig. 6b). As previously shown, *Lgr5* expression re-appeared in the crypt bottom 48 h after ablation^25^, yet not at the villus tip, as evident by the complete loss of GFP signal (Fig. 6c) and Lgr5 mRNA (Supplementary Fig. 5).

Ablation of *Lgr5*+ crypt base columnar cells has been shown to initiate a regenerative program in the remaining crypt cells^33^. Importantly, migration of epithelial cells from the crypt to the villus tip takes 3–5 days^1,3^. We therefore argued that at short times following ablation of *Lgr5*+ cells, reduction in the expression of genes normally confined to the villus tip epithelium would be a result of loss of signals from the *Lgr5*+ VTTs, rather than incoming enterocytes, the state of which was perturbed in the crypt. To obtain a global view of the impact of VTTs on villus tip enterocyte gene expression, we therefore performed RNA sequencing of the epithelial layer 48 h after DT administration (Supplementary data 5, Fig. 6d, e, Methods). We identified a significant repression in enterocyte genes that were normally zonated towards the tip (Spearman correlation of *R* = −0.53, *p* < 10^−60^ 48 h post ablation between expression change following ablation and expression zone, Fig. 6e, Supplementary data 6). Villus tip enterocyte genes that were strongly reduced in expression upon VTT ablation included the purine metabolism module genes *Slc28a2*, *Nt5e*, and *Ada* (Fig. 6d–i).

Additional prominent villus tip enterocyte genes include the transcription factors *Klf4* and *Fos*, *Cdh1*, encoding E-cadherin and *Egfr*^4^. These genes have a relatively high basal expression level either throughout the villi axis (*Cdh1*, *Klf4*, and *Fos*) or at the crypt (*Egfr*). Expression changes in enterocyte at the villus tip could thus be masked for such genes in bulk RNA sequencing (Fig. 6d). To assess their expression changes, we therefore performed smFISH and measured expression specifically at the tip enterocytes, identifying a significant reduction of *Egfr*, *Cdh1*, *Klf4*, and *Fos*, in addition to *Ada*, *Nt5e* and *Slc28a2* (Fig. 6f–i). *Creb3l3*, an enterocyte gene that is elevated at the villi tip, did not exhibit changes in expression upon VTT ablation. Epithelial Axin2 expression did not change following VTT ablation (Supplementary data 5). These results demonstrate that VTTs are important regulators of the spatial expression programs of enterocytes at the villus tip, instructing the epithelial expression of key genes such as *Egfr*, *Cdh1*, *Klf4, Fos*, *Nt5e*, *Ada,* and *Slc28a2*.

**Fig. 7:**
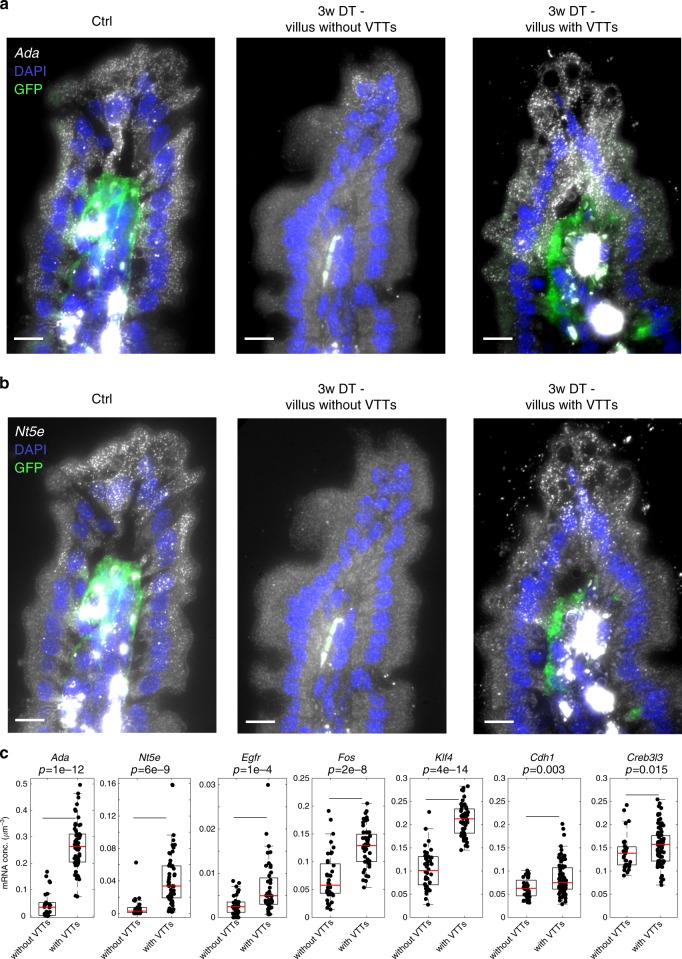
Analysis of the villus tip epithelial cells of *Lgr5–GFP-DTR* 3 weeks after VTT ablation shows correlation between the re-appearance of VTTs and the expression of enterocyte tip genes. **a** smFISH example of *Ada* showing higher expression in the villus tip with VTTs compared to villus tip without VTTs after 3 weeks. Scale bar 10 µm. **b** smFISH example of *Nt5e* showing higher expression in the villus tip with VTTs compared to villus tip without VTTs after 3 weeks. Scale bar 10 µm. **c** Quantification of expression differences between villi with VTTs and villi without VTTs. Measurements were performed over 15 villi per mouse for two mice. Indicated *p* values were calculated by two-sided Wilcoxon rank sum test. Boxes show 25–75 percentiles of the smFISH expression, horizontal red lines are medians, whiskers extend to the most extreme data point within 1.5× the interquartile range (IQR) from the box.

To assess the long-term consequences of VTT ablation we examined small intestinal tissue three weeks after VTT ablation. At this time point, VTTs re-appeared in 65% of the villi tips (Fig. 7, 46 GFP+ villi out of 71 villi, counted over 2 mice). Notably, the mRNA levels of the enterocyte villus tip genes *Ada*, *Nt5e*, *Egfr*, *Fos,* and *Klf4* remained significantly lower in the villi tips that lacked VTTs, compared to tips where VTTs reappeared (Fig. 7, mRNA conc. more than 2-fold higher in GFP+ vs. GFP− villi). In contrast, *Cdh1* and *Creb3l3* exhibited only a slight, yet statistically significant higher expression in the GFP+ villi (1.2 fold and 1.13 fold for *Cdh1* and *Creb3l3*, respectively, in GFP+ vs. GFP−). The correlation between VTT re-appearance and villus tip epithelial gene expression at this time point, when crypts have already returned to normal homeostatic state, further supports the instructive role of VTTs in regulating the enterocyte villus tip expression program.

## Discussion

Our study exposed the spatial diversity of mesenchymal cells along the crypt-villus axis. We identified a cell population of *Lgr5*+ VTTs that form a highly localized niche for the villus tip epithelium, facilitating the massive transcriptional changes that enterocytes undergo before they are shed from the tissue^4^. VTTs are a source of Bmp ligands (Fig. 2d, e, Supplementary Fig. 4c, Supplementary data 1) and may implement a switch from canonical to non-canonical Wnt signaling, through the expression of the non-canonical Wnt ligand *Wnt5a* (Fig. 5b). Similar spatially antagonistic expression of stromal *Wnt5a* expression and canonical Wnt activity has been recently demonstrated in the mouse prostate^31^. This switch may be important for the induction of E-cadherin, a major component of the adherens junction that is strongly induced at the villus tip enterocytes (Fig. 5d)^34^. *Wnt5a* is also an activator of the AP1 transcription factors^35^, composed of the enterocyte villus tip genes Fos and Jun^4^. VTTs also express components of the ECM, such as microfibrillar proteins (Fig. 5a), and therefore their ablation could result in perturbed ECM composition, indirectly affecting enterocyte gene expression. Future work, involving VTT-specific ablation of signaling components such as *Wnt5a* and Bmp ligands will further resolve the molecular mechanisms involved in the induction of specific enterocyte villus tip genes by VTTs.

VTTs are ideally suited to orchestrate gene expression programs along the intestinal villi. Their close proximity to the epithelial sheet and their intricate extensions towards the lamina propria facilitate close interactions with both epithelial and other stromal cell types. Unlike the short-lived epithelial cells and the often mobile immune cells, VTTs are static, quiescent and long-lived, and can therefore integrate long-term information related to diets and pathologies. The intestine exhibits a remarkable ability to adapt to such perturbations^36,37^. Future work will resolve the roles of VTTs in shaping villus epithelial programs in such perturbed states. The re-appearance of VTTs 3 weeks following their ablation (Fig. 7) supports their importance for preserving the normal epithelial function. It will be important to identify the cellular origins of these re-appearing VTTs. Our study exposed VTTs as major regulators of the expression of villus tip enterocyte genes (Fig. 6e), yet enterocyte gene expression changes throughout the villus axis^4^. It will be interesting to explore the additional effects of zonated luminal signals and potential enterocyte ‘internal clocks’ in shaping enterocyte zonation. This could be achieved by modulating diets and microbial composition, or by modulating enterocyte turnover, and consequently migration rates, respectively.

Lgr5+ fibroblasts have previously been identified in the lung^38^, suggesting that *Lgr5*+ stromal cells may constitute a localized mesenchymal niche component in other tissues. It will be important to use similar spatial transcriptomics approaches to explore the cross-talk between the mesenchymal cell populations and zonated immune cell populations^26^, as well as other stromal components such as enteric neurons^27^. Our finding of positive expression of the *Lgr5-EGFP-IRES-creERT2* knock-in, as well as the *Lgr5–GFP-DTR* knock-in cassettes at the villus tip is important for interpreting physiological effects of perturbations driven by the Lgr5 promoter, such as lineage tracing, cell ablation and conditional knock-outs.

Recent efforts for establishing tissue cell atlases rely on the ability to deconvolve the complex spatial interactions of distinct parenchymal and non-parenchymal cell types in each tissue^13^. Our study performs such combined analysis in the mouse small intestine, a prototypical zonated tissue. Similar approaches could be applied to reveal zonated interactions between stroma and epithelial cells in other structured organs.

## Methods

### Mice and tissues

All animal studies were approved by the Institutional Animal Care and Use Committee of WIS and UCSF. C57bl6 male mice age 8-10 weeks were obtained from the Harlan laboratories, all mice were fed regular chow ad libitum. All experiments were performed on the same region of the Jejunum, measured as 8 cm from the stomach-Duodenum boundary (Lgr5+ VTTs appear throughout the small intestine, data not shown). Mice were sacrificed by cervical dislocation. For LCM, jejunum tissues were harvested and embedded directly in OCT without fixation. For smFISH, jejunum tissues were harvested and fixed in 4% formaldehyde for 3 h, incubated overnight with 30% sucrose in 4% formaldehyde and then embedded in OCT in the form of swiss-rolls. Seven micrometer cryosections were used for hybridization. Mouse jejunum cells for single-cell RNAseq were extracted from eight mice. All smFISH quantifications were performed on at least 2 mice. Images in Fig. 3c, d  were taken from jejunum tissues of *Lgr5-EGFP-IRES-CreERT2* mice^2^. *Lgr5*^*DTRGFP*^ (*Lgr5-GFP-DTR*) mice^25^ were housed in the University of California San Francisco (UCSF) animal facility in compliance with all ethical guidelines established by the Institutional Animal Care and Use Committee (IACUC) and Laboratory Animal Resource Center.

### Antibodies used in this study

The following antibodies were used for FACS cell isolation: CD31 (PE-Cy7 102418, Biolegend), CD45 (APC-Cy7 103116, PE-Cy7 103114, Biolegend), PDGFRa (Alexa 488 FAB1062G, R&D systems, APC 135908, Biolegend), EPCAM (PE 118205, Biolegend). All FACS antibodies were used at 1:100 dilution in the presence of TruStain fcX blocker (101320, Biolegend, 1:50). For PDGFRa immunofluorescence staining, goat anti mouse PDGFRa was used as first antibody (AF1062 R&D systems) at a concentration of 8 µg/µl in the smFISH hybridization buffer, Alexa fluor 488 conjugated Donkey anti goat (705-545-147 Jackson laboratories, 1:400) was used as secondary antibody. For E-CADHERIN immunofluorescence staining FITC anti E-CADHERIN (612131 BD Biosciences) was used at dilution of 1:100.

### Laser capture microdissection (LCM)

Tissue blocks for laser-capture microdissection were freshly prepared at the morning of the collection day. The jejunum part was transected on top of Wattman paper soaked in PBS to support the opened structure in petri dish on ice. The tissue was washed with cold PBS and embedded in OCT on dry ice. LCM protocol was applied as previously described^4,39^ with minor modifications. Sections of 9 µm thickness were cut from the frozen block, mounted on polyethylene-naphthalate membrane-coated 518 glass slides (Zeiss, 415190-9081-000), air-dried for 20 s at room temperature, washed in 70% ethanol for 25 s, incubated in water for 25 s (Sigma-Aldrich, W4502), stained with HistoGene Staining Solution for 20 s (ThermoFisher Scientific, KIT0401), washed again in water for 25 s. The stained sections were dehydrated with subsequent 25 s incubations in 70%, 95%, and 100% EtOH and air-dried for 60 s before microdissection. Tissue sections were examined using a bright field imaging microscope (Observer.Z1, Zeiss), and microdissected with a UV laser-based unit (PALM-Microbeam, Zeiss). To ensure minimal damage to the surrounding cells, laser intensity and focus were calibrated before each session using Zeiss calibration wizard supplemented with the LCM operating software (Zeiss). Manual detection of analyzed regions in each tested slide and labeling of the desired areas were done with PALM ×10 and ×20 lenses. Tissue fragments were catapulted and collected in 0.2 ml adhesive cap tubes (Zeiss, 415190-9191-000). Each capture section was visually confirmed by focusing the PALM on the targeted adhesive cap following the collection session and immediately resuspend in 10.5 µl of 1× reaction buffer from SMART-Seq v4 Ultra Low Input RNA Kit (Clontech, 634888). Tissue lysis was achieved by incubation for 5 min at room temperature and stored at −20 °C. Collected samples were stored at −80 °C until the library preparation. Three distinct zones spanning approximately equal heights from the crypt-villus border to the villus tip, were collected from long villi (about 500 µm)-villus bottom, villus center and villus top (Fig. 1). An area of ~30,000 μm^2^ per zone was collected for each mouse by pooling 9–11 distinct villi from a single tissue section. For mice 1–3 (Supplementary data 1) we additionally catapulted crypt stroma and extracted material from two sequential tissue sections for each zone, with the exception of the villus center zone of mouse 3, where one section failed to amplify (Supplementary data 1, TPM value tab).

### SMART-Seq for bulk LCM samples

RNA libraries from the bulk tissues were prepared using SMART-Seq v4 Ultra Low Input RNA Kit (Clontech, 634888), with a 15 PCR cycles for the amplification step. Subsequent steps were applied as mentioned in the protocol. Nextera XT DNA Library Prep Kit (Illumina) was use to finalize the libraries. Library concentration and quality control were determined using NEBNext Library Quant Kit (New England Biolabs) and Agilent High Sensitivity D1000 ScreenTape System (Agilent, 5067-5584). Library final concentration of 2.4pM was loaded on NextSeq550 (Illumina) sequencing machine aiming for 20 M reads per sample with the following cycle distribution: 38 bp read1, 8 bp index1, 8 bp index2, 38 bp read2.

### Stroma LCM bulk RNA-seq analysis

Illumina raw files were converted to FASTQ files using bcl2fastq 2.17 (Illumina), following by pseudoalignment with Kallisto 0.43.0^40^ to a transcriptome index of the GRCm38.94 (Ensembl), filtered to transcripts with “coding_genes” entry. The following flag was used for kallisto: --b 100 (the number of bootstrap samples), --rf-stranded (strand specific reads, first read reverse). Sleuth 0.30.0^41^ running on R 3.5.1 was utilized to create a TPM table (Transcripts Per Million) for each sample (Supplementary data 1).

### Stroma LCM data processing

To exclude transcripts that might have originated from residual enterocytes included in the laser microdissected tissue, we filtered out genes that were highly expressed in enterocytes. To this end, we considered the maximal expression of all genes in enterocytes over the zones as measured in Moor et al.^4^. We sorterd these genes by expression levels and removed 31 non-ribosomal and non-mitochondrial genes that were included within the list of highly expressed genes, the cumulative summed expression of which exceeded 50% of the total enterocyte expression. We normalized the TPM values upon removing these genes by the sum of all remaining TPM. We next divided the gene expression values in each sample by the sum for all genes that individually make up less than 1% of the sample’s summed expression values. For each of the four zones, we computed the means and standard errors of the means over the different samples. Ligand receptor pairs were extracted from Ramilowsky et al.^12^, Matrisome components were extracted from Naba et al.^17^. Only genes with maximal zonation value larger than 5*10^−6^ were considered.

### Analysis of Il18r1 stromal cells

We used the single cell RNAseq data of Biton et al.^26^ (6558 cells annotated as WT control, Supplementary Fig. 1c). Il18r1 positive cells belonged to a cluster annotated as ‘CD4’. We performed differential gene expression using Wilcoxon rank-sum tests between the *Il18r1*+ and *Il18r−* cells in that cluster (Supplementary Fig. 1d). We used Immgen My Geneset tab (https://www.immgen.org) to annotate the 200 genes that had the highest fold-change between *Il18r1*+ cells and *Il18r1−* cells within the CD4-annotated cluster (only genes with expression above 5 × 10^−5^ were used for this analysis). The gene set was enriched in innate lymphoid cells type 3.

### Hybridization and imaging

Probe libraries were designed using the Stellaris FISH Probe Designer Software (Biosearch Technologies, Inc., Petaluma, CA). 7 µm thick sections of fixed Jejunum were sectioned onto poly L-lysine coated coverslips and used for smFISH staining. The intestinal sections were hybridized with smFISH probe sets according to a previously published protocol^15^. Briefly, tissues were treated for 10 min with proteinase K (10 µg/ml Ambion AM2546) and washed twice with 2× SSC (Ambion AM9765). Tissues were incubated in wash buffer (20% Formamide Ambion AM9342, 2× SSC) for 5 min and mounted with the hybridization buffer (10% Dextran sulfate Sigma D8906, 20% Formamide, 1 mg/ml E.coli tRNA Sigma R1753, 2× SSC, 0.02% BSA Ambion AM2616, 2 mM Vanadyl-ribonucleoside complex NEB S1402S) mixed with 1:3000 dialution of probes. Hybridization mix was incubated with tissues for overnight in a 30°C incubator. SmFISH probe libraries (Supplementary data 7) were coupled to Cy5, TMR or Alexa594. After the hybridization, tissues were washed with wash buffer containing 50 ng/ml DAPI for 30 min at 30 °C. Telocytes cells were detected by PDGFRα primary antibody that was added to the smFISH hybridization buffer and Alexa fluor 488 conjugated Donkey anti goat as secondary antibody in GLOX buffer for 20 minutes after DAPI (Sigma-Aldrich, D9542) nuclear staining. All images were taken as scans extending from villus tip to crypt bottom using ×100 magnifications, hence several fields of view were stitched together to cover the whole crypt-villus unit. Stitching was performed with the fusion mode linear blending and default settings of the pairwise stitching plugin^42^ in Fiji^43^. Quantification of smFISH was done using ImageM^44^. For smFISH quantifications, results were based on at least 30 cells from each region and from at least 2 mice. Dots were counted in the first 3 μm of the Z-stack, and divided by the segmented cell volume to obtain the mRNA concentration per cell. Two-sided Wilcoxon rank-sum tests were used to assess significance. Quantifications of the proportions of *Lgr5*+ VTTs positive for the EGFP knock-in were done on jejunum tissues of *Lgr5-EGFP-Ires-CreERT2* mice^2^.

### Statistics and reproducibility

All smFISH micrographs are representative of at least 10 images and two mice per gene or region.

### Single cell isolation

Due to the difficulties of isolating intestinal stromal cells we applied several dissociation protocols and surface marker staining to obtain a broad sampling of cells. For all mice, cells were isolated from the jejunum. The jejunum was extracted and rinsed in cold PBS. The tissue was opened longitudinally and sliced into small fragments roughly 2 cm long and incubated in 10 mM EDTA-PBS on ice for 10 min. The tissue was then moved to warm 5 ml 10 mM EDTA-PBS containing Liberase TM (100 μg/mL, Sigma) and DNaseI (2 U/mL, Sigma) and incubated at 37 °C for 20 min while shaken vigorously every few minutes. At the end of the incubation time, 5 ml of cold PBS was added to the cell suspension. The supernatant was filtered through a 100 μm filter and centrifuged at 300 × *g* for 5 min. the pellet was resuspended in FACS buffer (2 mM EDTA, 0.5% BSA in PBS) and stained with the required antibodies for flow cytometry sorting.

Isolation of telocytes was performed as previously decribed in ref. ^9^. Briefly, Jejunum were dissected and washed thoroughly with Hank’s balanced salt solution (HBSS) and were incubated in 5 mM EDTA in HBSS for 10 min at 4 °C. Intestinal villi were scraped off using a coverslip and the remaining tissue was cut into small pieces and incubated in 5 mM EDTA and HBSS on ice for 10 min while pipetting to completely remove the remaining epithelium. After vigorous washes, the remaining mesenchymal fraction was incubated with 6 mg/ml Dispase II/0.05% trypsin solution (Sigma-Aldrich, 04942078001) supplemented with 1 U/ml DNaseI (Sigma) at 37 °C, until the solution became cloudy and the mesenchyme was dissociated (8 min). At the end of the incubation time, 3 ml of warm FCS were added to the cell suspension to stop the digestion. Supernatant was filtered through a 70-μm strainer, centrifuged at 2500 × *g* for 5 min. the pellet was resuspended in FACS buffer (2 mM EDTA, 0.5% BSA in PBS) and stained with the required antibodies for flow cytometry sorting.

### Single-cell sorting

Single cells were sorted with SORP-FACSAriaII machine (BD Biosciences) using a 100 μm nozzle. Dead cells were excluded on the basis of 500 ng/ml Dapi incorporation. Sorted cells were negative for CD45 and EPCAM and positive for PDGFRa. Cells were sorted into 384-well cell capture plates containing 2 μl of lysis solution and barcoded poly(T) reverse-transcription (RT) primers for single-cell RNA-seq^28^. Barcoded single cell capture plates were prepared with a Bravo automated liquid handling platform (Agilent) as described previously^28^. Four empty wells were kept in each 384-well plate as a no-cell control during data analysis. Immediately after sorting, each plate was spun down to ensure cell immersion into the lysis solution, snap frozen on dry ice and stored at −80 °C until processed.

### MARS-Seq library preparation

Single cell libraries were prepared, as described in^28^. Briefly, mRNA from cells sorted into MARS-Seq capture plates were barcoded and converted into cDNA by reverse transcription reaction and pooled using an automated pipeline. The pooled sample was cleaned using 0.9X SPRI beads and then linearly amplified by T7 in vitro transcription. The resulting RNA was fragmented and converted into sequencing ready library by tagging the samples with pool barcodes and Illumina i7 barcode sequences during ligation, reverse transcription and PCR. Each pool of cells was tested for library quality and concentration was assessed as described in^28^. Machine raw files were converted to fastq files using bcl2fastq package, to obtain the UMI counts, reads were aligned to the mouse reference genome (GRCm38.84) using zUMI packge^45^ with the following flags that fit the barcode length and the library strandedness: -c 1-7, -m 8-15, -l 66, -B 1, -s 1, -p 16. This analysis resulted in 2217 cells.

### scRNAseq data processing

For each single cell and for each gene we first subtracted the estimated background expression. Background was calculated for each 384-well plate separately, as the mean gene expression in the four empty wells. After subtraction, negative values were set to zero. Next, cells with total UMI counts lower than 400 or higher than 8000 and total gene counts lower than 250 were removed. We used Seurat v2.3.4 package in R^29^ v3.5.3 to visualize and cluster the single cell RNAseq data (Fig. 4, Supplementary Fig. 3). Gene expression measurements (UMIs per gene) were normalized for each cell by the summed UMI, multiplied by a scale factor 10,000, and then log-transformed. To avoid undesired sources of variation in gene expression, we used Seurat to regress out cell-cell variation driven by total number of UMIs, and mitochondrial genes fraction. For detection of variable genes, we set a bottom cutoff of 0.25 and a top cutoff of 4 on the regressed log-transformed average gene expression, as well as a bottom cutoff of 0.5 on the dispersion. Cell clustering was based on PCA dimensionality reduction using the first 11 PCs, and a resolution value of 2.

We used cell type-specific markers to interpret the resulting 3 main clusters, Epcam was highly expressed in the epithelial cells clusters, Ptprc was highly expressed in the immune cells clusters and Pdgfra was highly expressed in the fibroblast cells clusters.

We next focused on the cell clusters that had an average expression of Pdgfra that was higher than 10^−4^ per cell (fraction of UMI counts). These included 329 cells. For this new set of cells, we re-ran Seurat with the following parameters; For detection of variable genes, we set a bottom cutoff of 0.25 and a top cutoff of 4 on the regressed log-transformed average gene expression, as well as a bottom cutoff of 1 on the dispersion. Cell clustering was based on PCA dimensionality reduction using the first 5 PCs, and a resolution value of 0.7. Marker genes were detected with Seurat FindAllMarkers function with parameters min.pct = 0.2, logfc.threshold = 0.25 (Supplementary data 4).

To identify zonation of the four mesenchymal cell types we examined the summed expression of concise sets of markers that were specific to each cell type. To select these markers, we included genes that had an average expression higher than 5 × 10^−6^ and were expressed in at least 10 cells in the respective cell type. We sorted these genes by the fold change of their expression levels compared to the maximal average expression in the other three mesenchymal cell types. We included the top 20 genes and further excluded genes for which the fold change ratio was lower than 5-fold. Differential gene expression between crypt telocytes and VTTs (Fig. 5a) considered all genes with average expression larger than 10^−5^ of cellular UMIs and more than 5 cells positive for the gene in at least one of the two populations. Storey’s method was used to compute *q*-values.

### *Lgr5+* cell ablation

For *Lgr5*+ cell ablation, male *Lgr5*^*DTRGFP*^ mice aged 4–5 months were administered 50 μg/kg diphtheria toxin (322326 Sigma) or saline vehicle intraperitoneally. Sample were collected 24 or, 48 h or 3 weeks post injection. For the 24 h time points, four DT-injected and two mock-injected mice were used for histological examination (Fig. 6). For the 48 h time point, four DT-injected and four mock-injected mice were used for histological examination, smFISH and epithelial cell sequencing. In addition, two DT-injected and two mock-injected mice were sacrificed 20 days after administration and used for histological examination and smFISH. For epithelial cell isolation, 3 cm from the proximal jejunum were extracted and rinsed in cold PBS. The tissue was opened longitudinally and sliced into small fragments roughly 2 cm long and incubated in 10 mM EDTA-PBS on ice for 20 min. The tissue was then moved to warm 5 ml 10 mM EDTA-PBS and incubated at 37 °C for 5 min while shaken vigorously every few minutes. At the end of the incubation time, 5 ml of cold PBS was added to the cell suspension. The supernatant was filtered through a 100 μm filter and centrifuged at 300 × *g* for 5 min. the pellet was snap frozen in liquid nitrogen and was processed for bulk RNA sequencing. For smFISH blocks, 5 cm from the distal jejunum was fixed immediately in 4% FA and processed as mentioned before.

### Bulk RNA sequencing of ablation experiment samples

Snap frozen cells were thawed into TRI reagent (sigma), RNA was isolated by Direct-zol RNA MiniPrep kit (Zymo research) according to the manufacturer instructions. RNA was processed by the mcSCRBseq protocol^46^ with minor modifications. RT reaction was applied on 10 ng of total RNA with a final volume of 10 µl (1× Maxima H Buffer, 1 mM dNTPs, 2 µM TSO* E5V6NEXT, 7.5% PEG8000, 20U Maxima H enzyme, 1 µl barcoded RT primer). Subsequent steps were applied as mentioned in the protocol. Library preparation was done using Nextera XT kit (Illumina) on 0.6 ng amplified cDNA. Library final concentration of 2.2pM was loaded on NextSeq 500 (Illumina) sequencing machine aiming for 20 M reads per sample. Raw files were converted to FASTQ files using bcl2fastq package, to obtain the UMI counts, fastq reads were aligned to the mouse reference genome (GRCm38.84) using zUMI packge^45^ with the following parameters RD1 16 bp, RD2 66 bp with a barcode (i7) length of 8 bp. UMI counts were processed with the edgeR package^47^. Our analysis included four intestinal samples of DT-injected mice and four from mock-injected, 48 h after injection (Supplementary data 5). We first filtered the genes by expression to maintain only genes with expression greater than 10^−5^ of the summed UMI counts of the sample in at least 2 samples and next ran the calcNormFactors function with default settings. We used the “robust” option in the glmQLFit function to robustly estimate the QL dispersion and glmQLFTest with default parameters to compute differential gene expression with Benjamini-Hochberg correction for multiple hypotheses (Supplementary data 6). Figure 6d shows the volcano plots of all genes with with maximal expression higher than 5 × 10^−6^ in at least one of the samples, as well as maximal zonation higher than 5 × 10^−6^ in Moor et al.^4^.

### Reporting summary

Further information on research design is available in the Nature Research Reporting Summary linked to this article.

## Supplementary information

Supplementary Information

Reporting Summary

Description of Additional Supplementary Files

Supplementary Data 1

Supplementary Data 2

Supplementary Data 3

Supplementary Data 4

Supplementary Data 5

Supplementary Data 6

Supplementary Data 7

## Data Availability

All data has been deposited in the GEO database with accession code GSE134479.

## Code availability

All codes used in this study are available upon request.
