# Supplementary material for: Lgr5+ telocytes are a signaling source at the intestinal villus tip
Source: Nat Commun. 2020 Apr 22;11:1936. doi: 10.1038/s41467-020-15714-x (PMC7176679; doi:10.1038/s41467-020-15714-x)
Supplement: Supplementary file 1 — Supplementary Information [file 41467_2020_15714_MOESM1_ESM.pdf]

Supplementary Information

Lgr5+ telocytes are a signaling center at the intestinal villus tip

Bahar Halpern et al.

Supplementary Figures

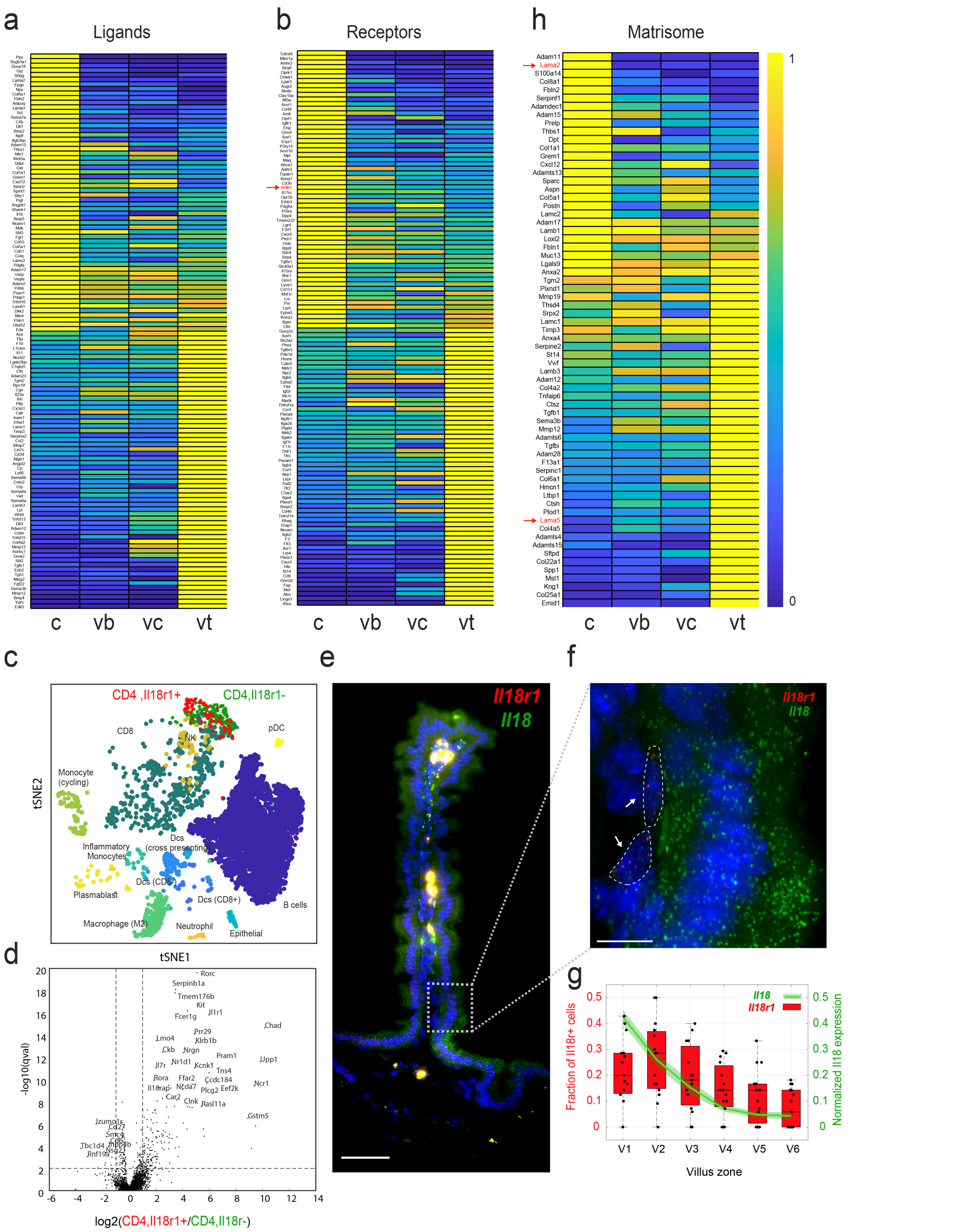

Supplementary Figure 1 – Zonated expression of stromal ligands (a) and receptors (b). *Il18r1* marked in red. c) tSNE plot of immune cells in the small intestine taken from Biton et. al.[1]. *Il18r1* is expressed in a subset of a cluster annotated as CD4 (red cells). d) Differential gene expression between the *Il18r1+* cells (red cells in c)) and *Il18r1-* cells of the same cluster (green cells in c)).

These genes are markers of innate lymphoid cells type 3. The y axis was truncated at qval=10^{-20}, leaving out *Il18r1* (having expression ratio of 4,240, qval=10^{-79}). Vertical lines denote log2 ratios of 1 and -1. Horizontal line denotes qvalue of 0.01. e) smFISH for *Il18* (green) and *Il18r1* (red) demonstrating spatial colocalization at the lower villus zones. Scale bar – 50µm. f) blow-up of a region boxed in e) demonstrating spatial adjacency of stromal immune cells with *Il18r1* transcripts (red dots, cells marked by red arrows and outlined in red) and *Il18+* villus bottom epithelial cells. Scale bar – 10 µm. g) Quantification of the fraction of *Il18r1+* cells (left red y-axis) along the villus axis (p values = 0.002, calculated by two-sided Wilcoxon rank sum test) showing colocalization to the region where *Il18* is highly expressed (right y-axis). *Il18* expression is taken from Moor et. al.[2], normalized to sum to 1 over villus zones. Measurements were performed over 15 villi from four mice. Boxes show 25-75 percentiles of the cells fractions, horizontal black lines are medians, whiskers, extend to the most extreme data point within 1.5× the interquartile range (IQR) from the box, each data point represent the fraction of cells in a specific villus zone across the different measured villi. h) Zonated expression of proteins constituting the extracellular matrix (ECM, also termed the 'matrisome'[3]). Red arrows highlight the crypt-zonated *Lama2* and villus tip-zonated *Lama5*, previously described to be inversely zonated[4,5]. Each row in (a,b,h) was scaled by its max expression across the four zones.

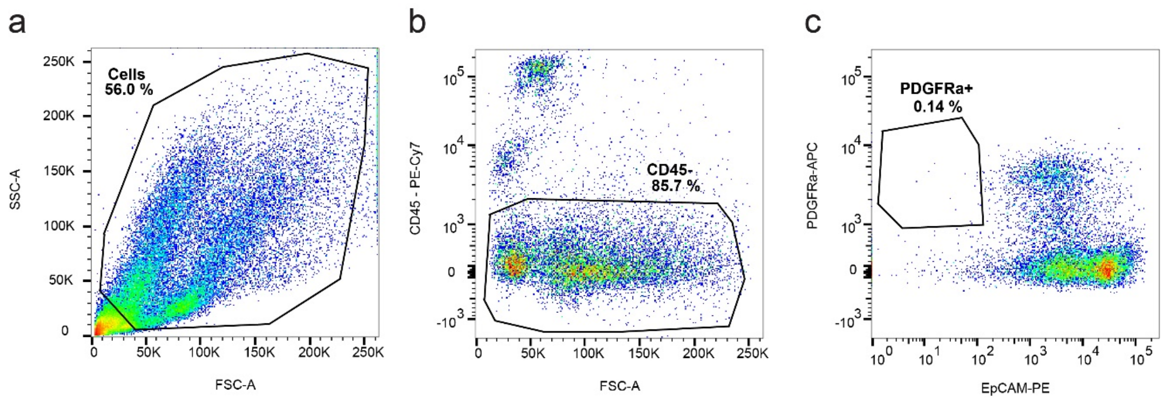

Supplementary Figure 2 – FACS gates used to enrich for PDGFRa+ cells that included the intestinal telocytes, as well as other mesenchymal cell types. a) FSC-A and SSC-A were used to select cells based on size. b) CD45 was used to gate out immune cells. c) PDGFRa and EPCAM fluorescense were used to select EPCAM negative, PDGFRa positive telocytes. Numbers in a-c) represent the percent of gated cells.

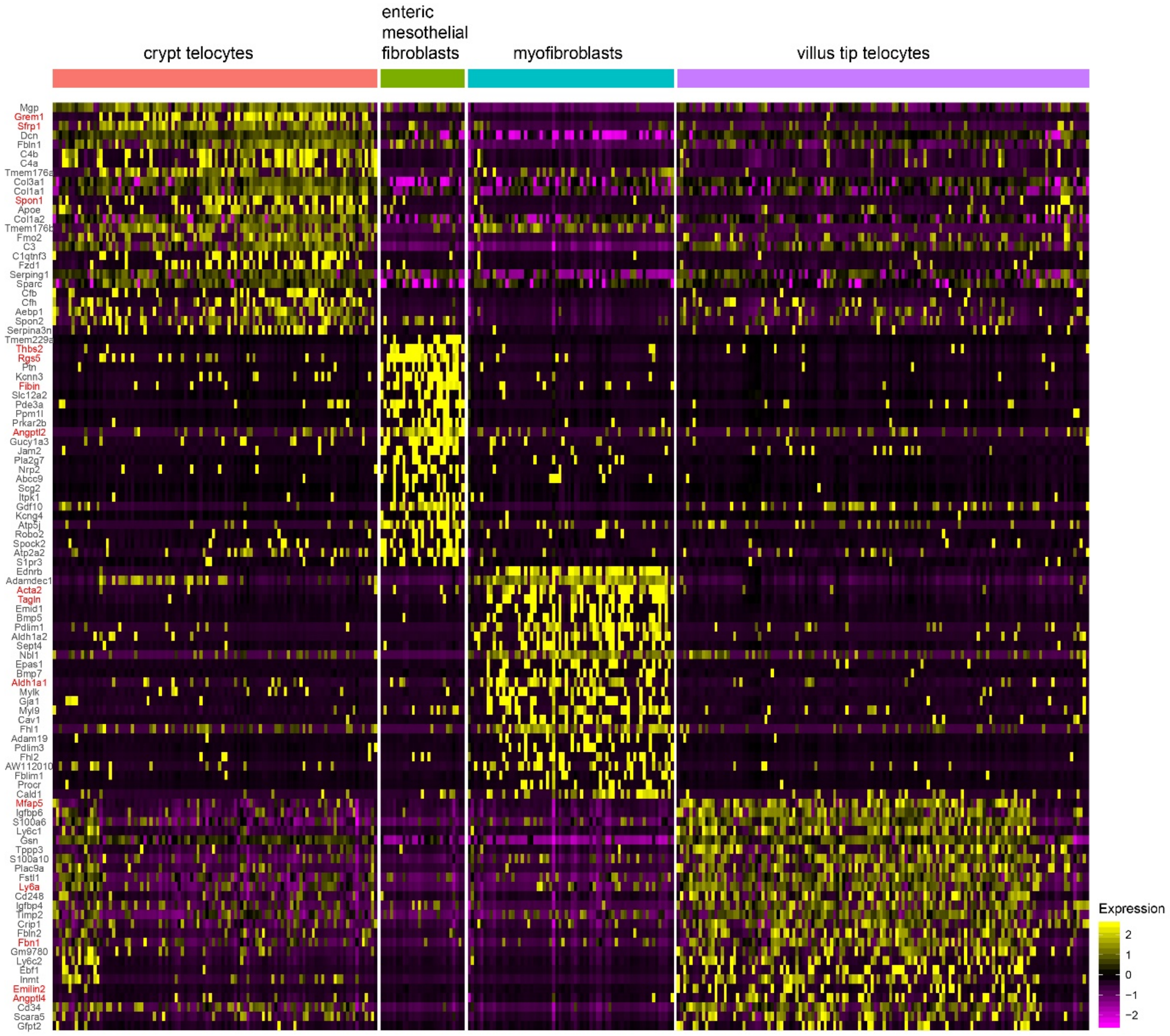

Supplementary Figure 3 – Heatmap showing the 25 top differentially expressed genes in each mesenchymal cell population - crypt telocytes, enteric mesothelial fibroblasts, myofibroblasts, villus tip telocytes. Genes discussed in the main text are marked in red.

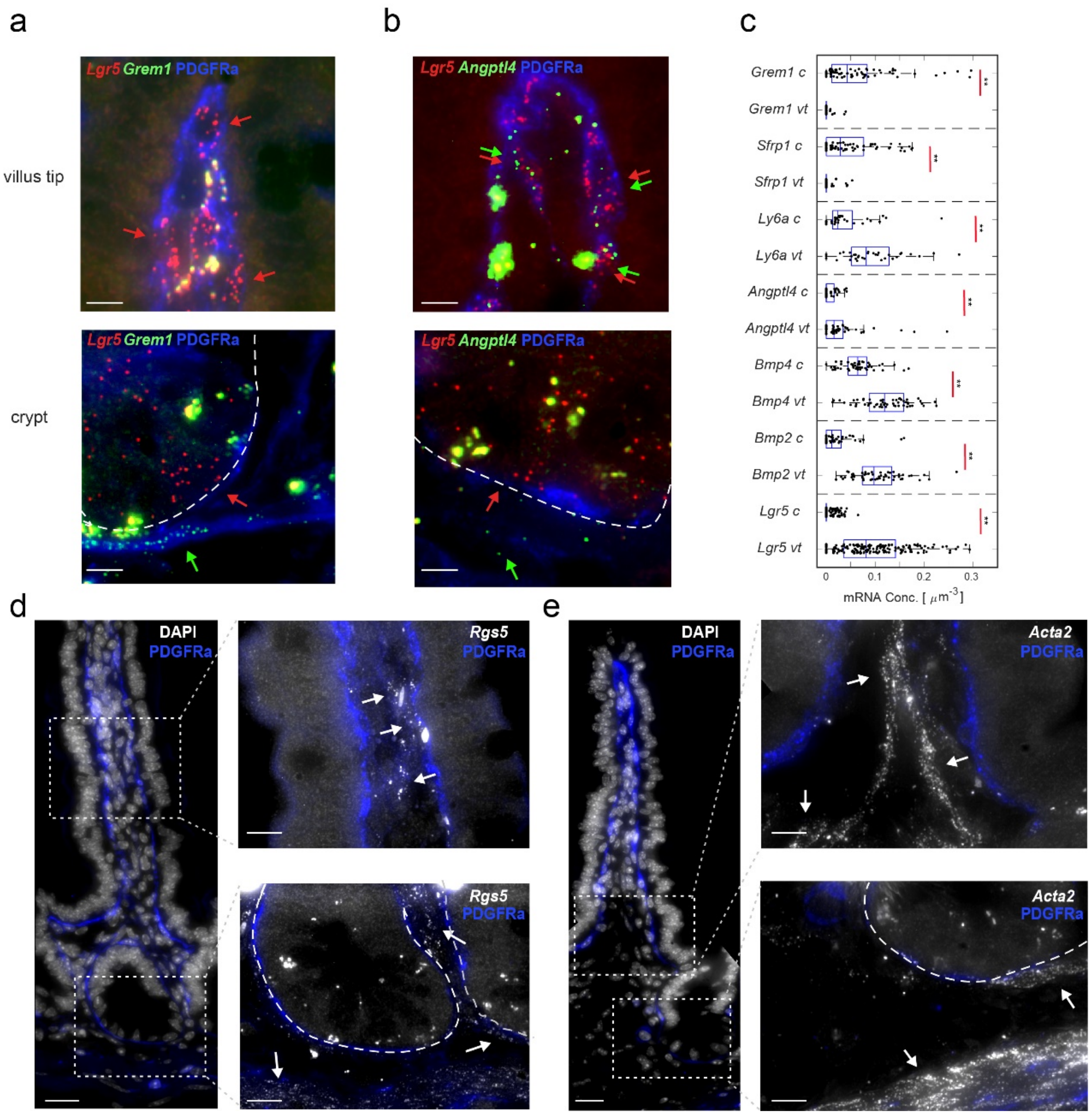

Supplementary Figure 4 – SmFISH validations of mesenchymal markers. a) *Grem1* mRNAs (green dots, green arrows mark positive cells) are expressed in crypt telocytes and not in VTTs. Scale bar – 5 µm. b) *Angptl4* (green dots) are expressed more highly in VTTs compared to crypt telocytes. Scale bar – 5 µm. Red dots are Lgr5 mRNAs (red arrows mark positive cells), blue is PDGFRa antibody staining. c) Quantification of smFISH validations for crypt telocytes and VTT markers. ** denote pvalue$<10^{-4}$ from two sided Wilcoxon ranksum test, c – crypt, vt – villus tip. Boxes show 25-75 percentiles of the smFISH expression, vertical lines are medians, whiskers, extend to the most extreme data point within 1.5× the interquartile range (IQR) from the box. d) Enteric mesothelial fibroblasts are scattered throughout the crypt-villus axis. Grey dots are mRNAs of *Rgs5*. blue is PDGFRa antibody staining. Scale bars – 20 µm, insets scale bar – 10 µm e) Myofibroblasts are scattered throughout the crypt-villus axis and localized away from the epithelial layer. Grey dots are mRNAs of *Acta2*. Blue is PDGFRa antibody staining. Scale bars – 20 µm, insets scale bar – 10 µm. White arrows mark positive cells in d) and e). White dashed line mark crypt borders.

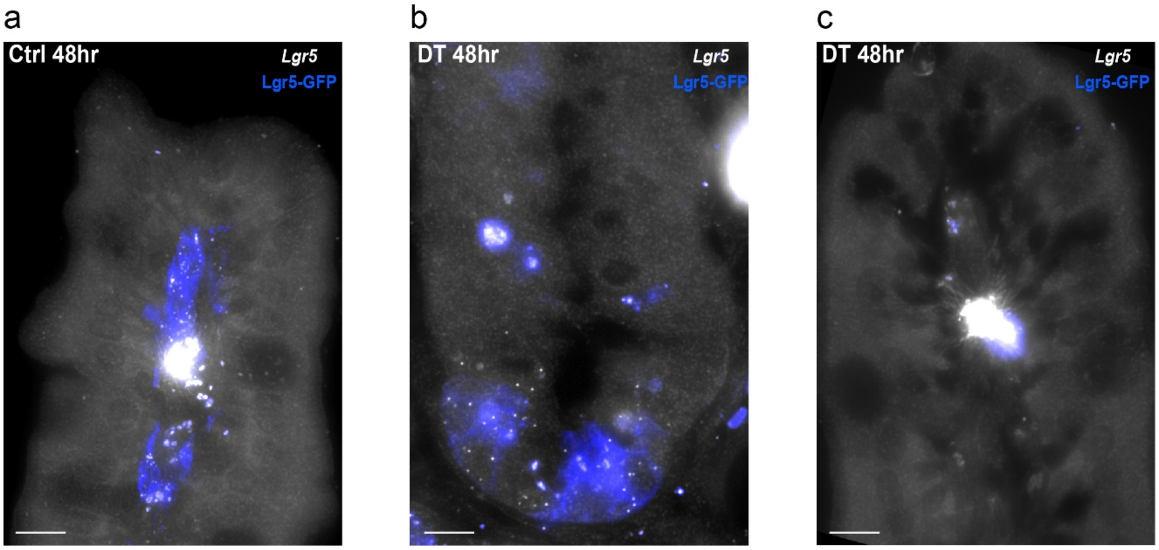

Supplementary Figure 5 – Villi tips are devoid of GFP and Lgr5 mRNA in Lgr5–GFP-DTR mice 48hr after VTT ablation. a) Bright dots are single mRNA of *Lgr5* co-localized with GFP positive cells. b) GFP and *Lgr5* reappear at the crypt stem cells. c) Villi tips are devoid of GFP and *Lgr5* mRNA. For a-c Scale bar - 10 µm.

Supplementary References

1. Biton, M. *et al.* T Helper Cell Cytokines Modulate Intestinal Stem Cell Renewal and Differentiation. *Cell* **175**, 1307-1320.e22 (2018).
2. Moor, A. E. *et al.* Spatial Reconstruction of Single Enterocytes Uncovers Broad Zonation along the Intestinal Villus Axis. *Cell* (2018) doi:10.1016/j.cell.2018.08.063.
3. Naba, A. *et al.* The extracellular matrix: Tools and insights for the "omics" era. *Matrix Biol.* **49**, 10–24 (2016).
4. Simon-Assmann, P., Spenle, C., Lefebvre, O. & Kedinger, M. The role of the basement membrane as a modulator of intestinal epithelial-mesenchymal interactions. *Prog. Mol. Biol. Transl. Sci.* **96**, 175–206 (2010).
5. Meran, L., Baulies, A. & Li, V. S. W. Intestinal Stem Cell Niche: The Extracellular Matrix and Cellular Components. *Stem Cells Int.* **2017**, (2017).
