# Supplementary material for: Lgr5+ telocytes are a signaling source at the intestinal villus tip
Source: Nat Commun. 2020 Apr 22;11:1936. doi: 10.1038/s41467-020-15714-x (PMC7176679; doi:10.1038/s41467-020-15714-x)
Supplement: Supplementary file 2 — Reporting Summary [file 41467_2020_15714_MOESM2_ESM.pdf]

nature research

Corresponding author(s): Shalev Itzkovitz, Michal Shoshkes-Carmel

Last updated by author(s): Feb 24, 2020

Reporting Summary

Nature Research wishes to improve the reproducibility of the work that we publish. This form provides structure for consistency and transparency in reporting. For further information on Nature Research policies, see Authors & Referees and the Editorial Policy Checklist .

Statistics

For all statistical analyses, confirm that the following items are present in the figure legend, table legend, main text, or Methods section.

n/a	Confirmed	
☐	☒	The exact sample size (n) for each experimental group/condition, given as a discrete number and unit of measurement
☐	☒	A statement on whether measurements were taken from distinct samples or whether the same sample was measured repeatedly
☐	☒	The statistical test(s) used AND whether they are one- or two-sided<br>*Only common tests should be described solely by name; describe more complex techniques in the Methods section.*
☒	☐	A description of all covariates tested
☒	☐	A description of any assumptions or corrections, such as tests of normality and adjustment for multiple comparisons
☐	☒	A full description of the statistical parameters including central tendency (e.g. means) or other basic estimates (e.g. regression coefficient) AND variation (e.g. standard deviation) or associated estimates of uncertainty (e.g. confidence intervals)
☐	☒	For null hypothesis testing, the test statistic (e.g. F, t, r) with confidence intervals, effect sizes, degrees of freedom and P value noted<br>*Give P values as exact values whenever suitable.*
☒	☐	For Bayesian analysis, information on the choice of priors and Markov chain Monte Carlo settings
☒	☐	For hierarchical and complex designs, identification of the appropriate level for tests and full reporting of outcomes
☒	☐	Estimates of effect sizes (e.g. Cohen's d, Pearson's r), indicating how they were calculated

Our web collection on statistics for biologists contains articles on many of the points above.

Software and code

Policy information about availability of computer code

Data collection	smFISH imaging was done on the NIS element software AR 5.11.01
Data analysis	Seurat 2.3.4, R3.5.3. Matlab 2018b, FIJI win64

For manuscripts utilizing custom algorithms or software that are central to the research but not yet described in published literature, software must be made available to editors/reviewers. We strongly encourage code deposition in a community repository (e.g. GitHub). See the Nature Research guidelines for submitting code & software for further information.

Data

Policy information about availability of data

All manuscripts must include a data availability statement. This statement should provide the following information, where applicable:

- Accession codes, unique identifiers, or web links for publicly available datasets
- A list of figures that have associated raw data
- A description of any restrictions on data availability

All data has been deposited in GEO with accession code GSE134479

Field-specific reporting

Please select the one below that is the best fit for your research. If you are not sure, read the appropriate sections before making your selection.

☒ Life sciences ☐ Behavioural & social sciences ☐ Ecological, evolutionary & environmental sciences

For a reference copy of the document with all sections, see nature.com/documents/nr-reporting-summary-flat.pdf

Life sciences study design

All studies must disclose on these points even when the disclosure is negative.

Sample size	The sample sizes (number of mice and total number of sequenced cells) where chosen as to be sufficient to obtain sufficient power to discern gene expression differences.
Data exclusions	single cell data was excluded on the basis of mitochondrial percentage, and minimum UMI and gene counts per cell in order to include only high quality live cells.
Replication	Each experiment was replicated n times (and n is given in each figure for each experiment). Although the exact quantitative results differ between replicates, the qualitative results were the same, so that it is reasonable to state that the "All attempts at replication were successful"
Randomization	For each experiment cage mates were taken on the same date. The mice were allocated randomly in this study
Blinding	The data collection was not blinded. Blinding was not possible as the investigators were also conducting the experiments and had to be aware of controls and treated groups.

Reporting for specific materials, systems and methods

We require information from authors about some types of materials, experimental systems and methods used in many studies. Here, indicate whether each material, system or method listed is relevant to your study. If you are not sure if a list item applies to your research, read the appropriate section before selecting a response.

Materials & experimental systems

n/a	Involved in the study	
☐	☒	Antibodies
☒	☐	Eukaryotic cell lines
☒	☐	Palaeontology
☐	☒	Animals and other organisms
☒	☐	Human research participants
☒	☐	Clinical data

Methods

n/a	Involved in the study	
☒	☐	ChIP-seq
☐	☒	Flow cytometry
☒	☐	MRI-based neuroimaging

Antibodies

Antibodies used	The following antibodies were used for FACS cell isolation: CD31 (PE-Cy7 102418, clone 390, Biolegend), CD45 (APC-Cy7 103116, clone 30-F11, PE-Cy7 103114, clone 30-F11, Biolegend), PDGFRa (Alexa 488 FAB1062G polycolnal,R&D systems, APC 135908, clone APA5, Biolegend), Epcam (PE 118205, clone G8.8, Biolegend). For PDGFRa immunofluorescence staining, goat anti mouse PDGFRa was used as first antibody (AF1062 polyclonal, R&D systems) at a concentration of 8 µg/µl in the smFISH hybridization buffer, Alexa fluor 488 conjugated Donkey anti goat (705-545-147 polyclonal,Jackson laboratories, 1:400) was used as secondary antibody. FITC anti E-Cadherin (612131 clone 36/E, BD Biosciences, 1:100)
Validation	all antibodies were validated on manufacturer website.<br>CD31 (PE-Cy7 102418, clone 390, Biolegend), for validation from website: "stain the endothelium of mouse lungs using the CD-31 antibody,The percentage of cells positive for CD31 were determined when sorting the cells". "The antibody stained the epithelium as expected".<br>CD45 (APC-Cy7 103116, clone 30-F11, PE-Cy7 103114, clone 30-F11, Biolegend), for validation from website: "in this experiment we are looking at populations of immune cells in the bone marrow of mice. This antibody is used to stain CD45 for flow cytometry". "This antibody is great for staining CD45".<br>PDGFRa (Alexa 488 FAB1062G polycolnal,R&D systems, APC 135908, clone APA5, Biolegend), antibody was used in Wang W, et al. 2014. Proc Natl Acad Sci U S A. 111:14466.<br>Epcam (PE 118205, clone G8.8, Biolegend). for validation from website: "In this experiment we were looking at type II alveolar epithelial cells in the lung of WT and KO mice", "This antibody works well for detecting EpCAM in the lung"<br>PDGFRa immunofluorescence staining, goat anti mouse PDGFRa was used as first antibody (AF1062 polyclonal, R&D systems) for validation from website: " PDGF R alpha was detected in immersion fixed frozen sections of mouse embryo using 15 µg/mL Goat Anti-Mouse PDGF R alpha Antigen Affinity-purified Polyclonal Antibody (Catalog # AF1062) overnight at 4 °C. "<br>FITC anti E-Cadherin (612131 clone 36/E, BD Biosciences, 1:100). antibody was used inMiyoshi K, Shillingford JM, Smith GH, et al. Signal transducer and activator of transcription (Stat) 5 controls the proliferation and differentiation of mammary alveolar epithelium. J Cell Biol. 2001; 155(4):531-542.

Animals and other organisms

Policy information about studies involving animals; ARRIVE guidelines recommended for reporting animal research

Laboratory animals	C57bl6 male mice age 8-10 weeks were obtained from the Harlan laboratories. male Lgr5DTRGFP mice aged 4-5 months were housed in the University of California San Francisco (UCSF) animal facility
Wild animals	no wild animals were used in this study
Field-collected samples	no field-collected samples were used in this study
Ethics oversight	All animal studies were approved by the Institutional Animal Care and Use Committee of WIS and UCSF.

Note that full information on the approval of the study protocol must also be provided in the manuscript.

Flow Cytometry

Plots

Confirm that:

- [x] The axis labels state the marker and fluorochrome used (e.g. CD4-FITC).
- [x] The axis scales are clearly visible. Include numbers along axes only for bottom left plot of group (a 'group' is an analysis of identical markers).
- [x] All plots are contour plots with outliers or pseudocolor plots.
- [x] A numerical value for number of cells or percentage (with statistics) is provided.

Methodology

Sample preparation	Due to the difficulties of isolating intestinal stromal cells we applied several dissociation protocols and surface marker staining to obtain a broad sampling of cells. For all mice, cells were isolated from the jejunum. The jejunum was extracted and rinsed in cold PBS. The tissue was opened longitudinally and sliced into small fragments roughly 2 cm long and incubated in 10mM EDTA-PBS on ice for 10 min. The tissue was then moved to warm 5ml 10mM EDTA-PBS containing Liberase TM (100 µg/mL, Sigma) and DNaseI (2 U/mL, Sigma) and incubated at 37°C for 20 min while shaken vigorously every few minutes. At the end of the incubation time, 5ml of cold PBS was added to the cell suspension. The supernatant was filtered through a 100µm filter and centrifuged at 300 g for 5 min. the pellet was resuspended in FACS buffer (2mM EDTA, 0.5% BSA in PBS) and stained with the required antibodies for flow cytometry sorting.<br>Isolation of telocytes was performed as previously decribed8. Briefly, Jejunum were dissected and washed thoroughly with Hank's balanced salt solution (HBSS) and were incubated in 5mM EDTA in HBSS for 10min at 4°C. Intestinal villi were scraped off using a coverslip and the remaining tissue was cut into small pieces and incubated in 5mM EDTA and HBSS on ice for 10min while pipetting to completely remove the remaining epithelium. After vigorous washes, the remaining mesenchymal fraction was incubated with 6mg/ml Dispase II/0.05% trypsin solution (Sigma-Aldrich, 04942078001) supplemented with 1U/ml DNaseI (Sigma) at 37°C, until the solution became cloudy and the mesenchyme was dissociated (8min). At the end of the incubation time, 3ml of warm FCS were added to the cell suspension to stop the digestion. Supernatant was filtered through a 70-µm strainer, centrifuged at 3500 rpm for 5 min. the pellet was resuspended in FACS buffer (2mM EDTA, 0.5% BSA in PBS) and stained with the required antibodies for flow cytometry sorting.
Instrument	SORP-FACSAriaII
Software	The software used to collect the data is FACSDiva.
Cell population abundance	The abundance of the Pdgfra+ cells in the population was 0.14%
Gating strategy	cells were gated using SSC and FSC gates (50K-250K for both). Next, dead cells were gated out based on DAPI incorporation. sorted cells were CD45 negative, EpCAM negative and PDGFRa positive.

- [x] Tick this box to confirm that a figure exemplifying the gating strategy is provided in the Supplementary Information.
