# Supplementary material for: Lgr5+ telocytes are a signaling source at the intestinal villus tip
Source: Nat Commun. 2020 Apr 22;11:1936. doi: 10.1038/s41467-020-15714-x (PMC7176679; doi:10.1038/s41467-020-15714-x)
Supplement: Supplementary file 3 — Description of Additional Supplementary Files [file 41467_2020_15714_MOESM3_ESM.pdf]

Description of Additional Supplementary Files

Supplementary Data 1

Average gene expression in the laser capture microdissected stromal zones. For each sample, UMI counts were normalized to the sum for all non-enterocyte genes that individually take up less than 1% of the total sample counts (Methods). TPM values presents the complete non-filtered data.

Supplementary Data 2

Correlations between the stromal and epithelial spatial expression domains of ligands and their respective receptors. Epithelial expression was taken from Moor et al.[4], Stromal expression taken from Table S1.

Supplementary Data 3

Average expression of the four mesenchymal cell populations based on the scRNAseq data. Expression is in units of fraction of total UMIs per cell.

Supplementary Data 4

Markers for the four mesenchymal cell populations obtained from Seurat[29]. P-value was calculated by two sided Wilcoxon ranksum test.

Supplementary Data 5

UMI counts for bulk RNAseq of epithelial cells extracted from DT-treated and mock-treated Lgr5–GFP-DTR mice 48 hrs after ablation.

Supplementary Data 6

Differential gene expression between epithelial cells extracted from DT-treated and mock-treated Lgr5–GFP-DTR mice 48 hrs after ablation. Two-sided p-value was calculated by the glmQLFtest R function.

Supplementary Data 7

Sequences of the smFISH probes libraries used in this study.
